# Paradoxical Patterns of Sinusoidal Obstruction Syndrome-Like Liver Injury in Aged Female CD-1 Mice Triggered by Cannabidiol-Rich Cannabis Extract and Acetaminophen Co-Administration

**DOI:** 10.3390/molecules24122256

**Published:** 2019-06-17

**Authors:** Laura E. Ewing, Mitchell R. McGill, Eric U. Yee, Charles M. Quick, Charles M. Skinner, Stefanie Kennon-McGill, Melissa Clemens, Joel H. Vazquez, Sandra S. McCullough, D. Keith Williams, Kristy R. Kutanzi, Larry A. Walker, Mahmoud A. ElSohly, Laura P. James, Bill J. Gurley, Igor Koturbash

**Affiliations:** 1 Department of Environmental and Occupational Health, University of Arkansas for Medical Sciences, Little Rock, AR 72205, USA; LEEwing@uams.edu (L.E.E.); MRMcgill@uams.edu (M.R.M.); CMSkinner@uams.edu (C.M.S.); SKennonmcgill@uams.edu (S.K.-M.); kristy.kutanzi@gmail.com (K.R.K.); 2 Department of Pharmacology and Toxicology, University of Arkansas for Medical Sciences, Little Rock, AR 72205, USA; MMClemens@uams.edu (M.C.); JVazquez@uams.edu (J.H.V.); McCulloughSandraS@uams.edu (S.S.M.); jameslaurap@uams.edu (L.P.J.); 3 Center for Dietary Supplements Research, University of Arkansas for Medical Sciences, Little Rock, AR 72205, USA; GurleyBillyJ@uams.edu; 4 Department of Pathology, University of Arkansas for Medical Sciences, Little Rock, AR 72205, USA; EYee@uams.edu (E.U.Y.); QuickCharlesM@uams.edu (C.M.Q.); 5 Department of Pediatrics, University of Arkansas for Medical Sciences, Little Rock, AR 72205, USA; 6 Department of Biostatistics, University of Arkansas for Medical Sciences, Little Rock, AR 72205, USA; WilliamsDavidK@uams.edu; 7 National Center for Natural Products Research, University of Mississippi, University, MS 38677, USA; lwalker@olemiss.edu (L.A.W.); melsohly@olemiss.edu (M.A.E.); 8 ElSohly Laboratories, Inc. (ELI), Oxford, MS 38677, USA; 9 Department of Pharmaceutics and Drug Delivery, School of Pharmacy, University of Mississippi, University, MS 38677, USA; 10 Department of Pharmaceutical Sciences, University of Arkansas for Medical Sciences, Little Rock, AR 72205, USA

**Keywords:** acetaminophen, cannabidiol, liver injury, natural products, phytochemical, sinusoidal obstruction syndrome

## Abstract

The goal of this study was to investigate the potential for a cannabidiol-rich cannabis extract (CRCE) to interact with the most common over-the-counter drug and the major known cause of drug-induced liver injury–acetaminophen (APAP)–in aged female CD-1 mice. Gavaging mice with 116 mg/kg of cannabidiol (CBD) [mouse equivalent dose (MED) of 10 mg/kg of CBD] in CRCE delivered with sesame oil for three consecutive days followed by intraperitoneally (i.p.) acetaminophen (APAP) administration (400 mg/kg) on day 4 resulted in overt toxicity with 37.5% mortality. No mortality was observed in mice treated with 290 mg/kg of CBD+APAP (MED of 25 mg/kg of CBD) or APAP alone. Following CRCE/APAP co-administration, microscopic examination revealed a sinusoidal obstruction syndrome-like liver injury–the severity of which correlated with the degree of alterations in physiological and clinical biochemistry end points. Mechanistically, glutathione depletion and oxidative stress were observed between the APAP-only and co-administration groups, but co-administration resulted in much greater activation of c-Jun N-terminal kinase (JNK). Strikingly, these effects were not observed in mice gavaged with 290 mg/kg CBD in CRCE followed by APAP administration. These findings highlight the potential for CBD/drug interactions, and reveal an interesting paradoxical effect of CBD/APAP-induced hepatotoxicity.

## 1. Introduction

Cannabidiol (CBD) is one of the major phytocannabinoids in *Cannabis sativa* that has seen a dramatic increase in popularity over recent years due to expanded availability and aggressive marketing. Though CBD is regarded as lacking psychotropic activity, it does display a number of neuropharmacological effects, including anti-seizure activity. CBD, as the prescription drug EPIDIOLEX®, recently received the US Food and Drug Administration (FDA) approval, with indications for treatment of certain severe forms of epilepsy [1,2]. Although that makes it a regulated drug in some forms, it is still widely marketed in an array of over-the-counter products in variable forms, and touted as a treatment for various medical conditions ranging from depression to arthritis and cancer [3,4].

Emerging evidence, however, indicates that CBD poses a significant risk for hepatotoxicity. A number of animal studies have reported increased liver weights, elevation of liver enzymes and bilirubin in circulation as well as molecular signatures of hepatotoxicity after administration of CBD or cannabidiol-rich cannabis extract (CRCE) [5,6,7]. In clinical trials, elevated liver enzymes were observed in 5–20% of patients treated with CBD, and several patients were withdrawn due to the threat of liver failure [1,2,8].

Evolving evidence suggests that CBD has the potential to induce drug interactions, involving the modulation of various cytochrome P450 (CYP) and UDP-glucuronosyltransferase (UGT) enzymes responsible for xenobiotic metabolism [7,9,10,11]. CBD was shown to modulate CYP2E1 and CYP1A2, two of the more common P450 enzymes involved in drug metabolism and drug-induced hepatotoxicity (e.g., acetaminophen (APAP) and ethanol). Such an interaction potential is especially concerning among the elderly, as CYP activity and drug clearance are known to be impaired with age [12,13]. Furthermore, rates of drugs use increase with age, with the highest overall prevalence observed in older women, of whom 94% take at least 1 medication, and 57% take 5 or more medications [14]. Lastly, recent research indicates that herbal dietary supplements pose a greater risk for hepatotoxicity among women (with similar patterns observed in female mice) [15,16,17]. Therefore, the goal of this study was to investigate the CBD/APAP interaction potential in aged female CD-1 mice.

## 2. Results and Discussion

### 2.1. Overt Toxicity Associated with CRCE/APAP Administration

Female, 9 month old, CD1 mice were gavaged over three consecutive days with vehicle, 116, or 290 mg/kg CBD. On the fourth day, mice were injected with either PBS or 400 mg/kg APAP. Three of the mice treated with 116 mg/kg CBD + APAP (*n* = 8) succumbed within 5 h of APAP dosing. No mortality was observed in the 290 mg/kg CBD+APAP or Veh+APAP mice. Mice in the CBD+APAP groups developed a sub-lethargic condition which was characterized by a substantial decrease in activity, nuzzling into bedding material, impaired response to external stimuli, and a decrease in body temperature. Veh+APAP mice developed hypothermia only.

Fluctuations in body weights were observed in mice among all experimental groups; however, in 116 mg/kg CBD+APAP mice there were statistically significant decreases in body weights (−4%, *p* < 0.05) (Supplementary Figure S1A). Furthermore, significant increases in liver-to- body weight (LBW) ratios were observed in 116 mg/kg CBD + APAP (*p* < 0.01) (Supplementary Figure S1B). No significant differences were observed in heart-or kidney-to-body weight ratios (Supplementary Figure S1C,D).

### 2.2. CRCE/APAP Cause Acute Liver Injury

**Figure 1:**
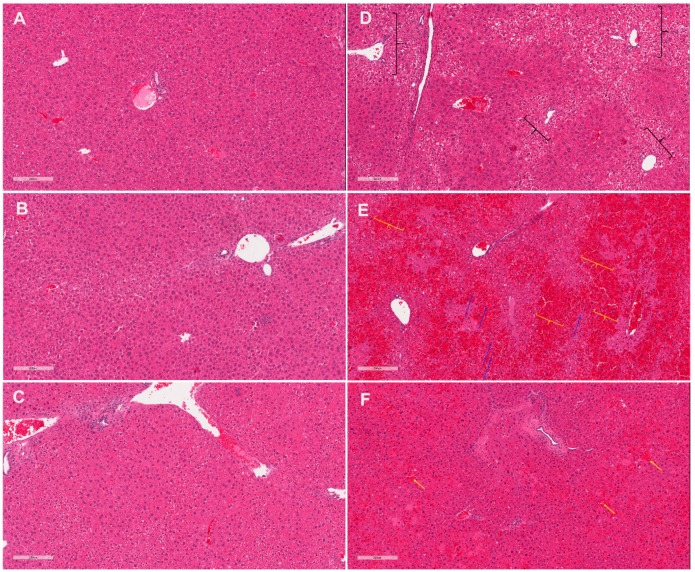
Co-administration of cannabidiol-rich cannabis extract (CRCE) and APAP results in sinusoid obstructive syndrome-like histomorphological alterations in the livers of aged female CD-1 mice. H&E stained liver sections from (**A**) vehicle-gavaged mice (sesame oil), (**B**) 116 mg/kg CBD, (**C**) 290 mg/kg CBD, (**D**) APAP (400 mg/kg), (**E**) 116 mg/kg CBD+APAP, (**F**) 290 mg/kg CBD+APAP. Black brackets indicate areas of clear cell changes (**D**), yellow brackets (**E**) and arrows (**F**) indicate areas of sinusoidal dilation with vascular congestion, and blue arrows (**E**) indicate areas of hepatic plate atrophy. CRCE—cannabidiol-rich cannabis extract; APAP—acetaminophen. Magnification: X10.

Histopathological evaluation of liver samples revealed abnormalities in the 116 mg/kg CBD+APAP mice manifested as a sinusoidal obstruction syndrome, or SOS, pattern of injury (Figure 1). Specifically, diffuse, centrivenular-dominant regions of sinusoidal dilation accompanied by marked vascular congestion, hepatic plate atrophy, and centrivenular necrosis were observed in the livers of six mice in this group (Table 1). However, foci of sinusoidal destruction were observed in the livers of only two out of eight mice in 290 mg/kg CBD + APAP and one out of seven mice in the Veh+APAP group.

**Figure 2:**
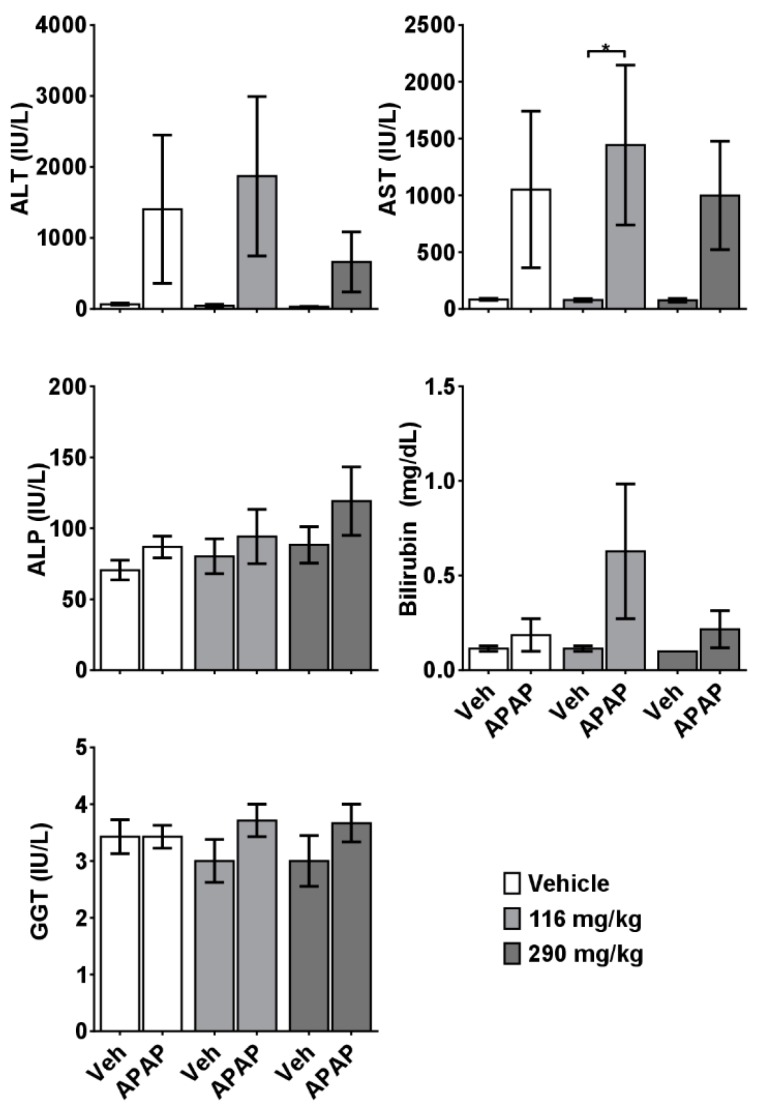
Effects of CRCE/APAP co-administration on commonly assessed clinical chemistry parameters for liver injury. Blood was collected at the time of sacrifice, and the serum was subsequently separated and removed for analysis. Veh (*n* = 7); Veh + APAP (*n* = 7); 116 mg/kg CBD (*n* = 7); 116 mg/kg CBD+APAP (*n* = 5); 290 mg/kg CBD (*n* = 7); 290 mg/kg CBD + APAP (*n* = 6). Data are presented as mean ± SEM (* *p* < 0.05). ALT: alanine aminotransferase; AST: aspartate aminotransferase; ALP: alkaline phosphatase; GGT: gamma-glutamyl transferase.

No significant differences were observed in the serum levels of total bilirubin and AST in experimental mice. This was likely due, in part, to the fact that serum samples were not available from deceased animals in the 116 mg/kg CBD + APAP group, nor from several moribund mice whose condition precluded adequate blood volume withdrawal. However, elevated serum levels of ALT were observed in 116 mg/kg CBD+APAP mice, compared to the control mice (Figure 2), and the extent of alterations in physiological (body weight, LBW) and clinical biochemistry endpoints significantly correlated with the degree of liver injury (Table 2).

### 2.3. Molecular Alterations Associated with CRCE/APAP

**Figure 3:**
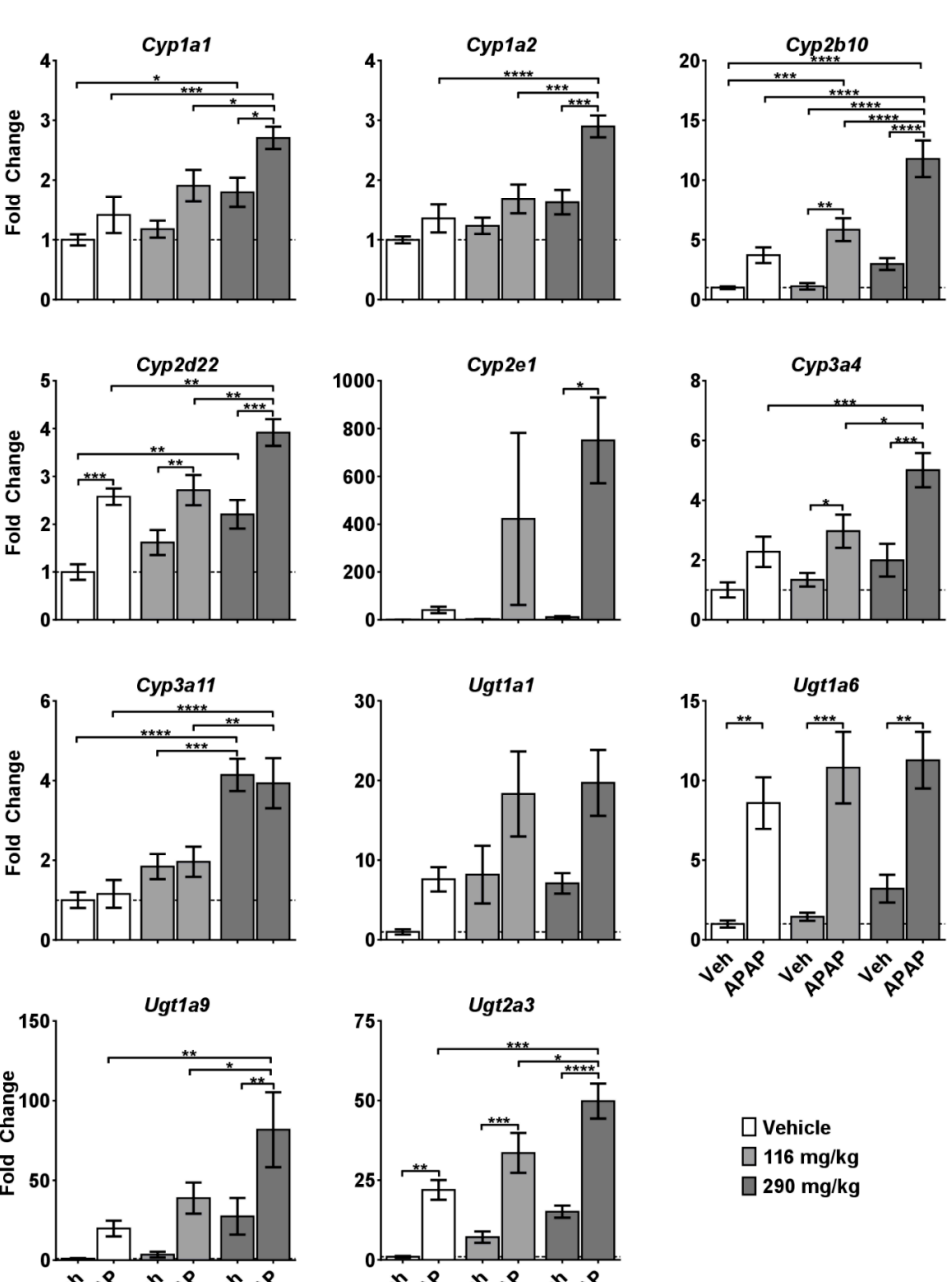
Effects of CRCE/APAP co-administration on intrahepatic expression of cytochrome P450s and UDP-glucuronosyltransferases. Livers were collected 5 h after APAP administration and gene expression was measured using the quantitative real-time (qRT) PCR. Veh (*n* = 7); Veh + APAP (*n* = 7); 116 mg/kg CBD (*n* = 7); 116 mg/kg CBD + APAP (*n* = 7); 290 mg/kg CBD (*n* = 8); 290 mg/kg CBD + APAP (*n* = 8). Data presented as mean ± SEM fold changed from vehicle (* *p* < 0.05; ** *p* < 0.01; *** *p* < 0.001; and **** *p* < 0.0001). The following genes were changed significantly with either CBD or APAP, but the two-way ANOVA did not yield any significant comparisons: *Cyp2d22* (APAP, *p* = 0.0016), *Ugt1a1* (CBD, *p* = 0.0186; APAP, *p* = 0.0255), *Ugt1a6* (CBD, *p* = 0.0222), and *Ugt1a9* (APAP, *p* = 0.0026).

To gain insight into the mechanisms of CBD-induced exacerbation of APAP hepatotoxicity, we first measured expression of a panel of genes involved in the metabolism of CBD and APAP. APAP itself did not cause substantial changes in mRNA levels in any of the investigated genes, except for *Ugt1a6* and *Ugt2a3* transferases (Figure 2). However, dose-dependent increases in the expression of numerous CYPs and UGTs were observed in the CBD treated mice. Furthermore, the CBD + APAP group had increased expression of *Cyp2b10*, and a strong interaction effect was observed in the case of *Cyp2e1* after 290 mg/kg CBD + APAP, but not after 116 mg/kg CBD + APAP (Figure 3).

### 2.4. Mechanisms of CRCE/APAP-Induced Liver Injury

**Figure 4:**
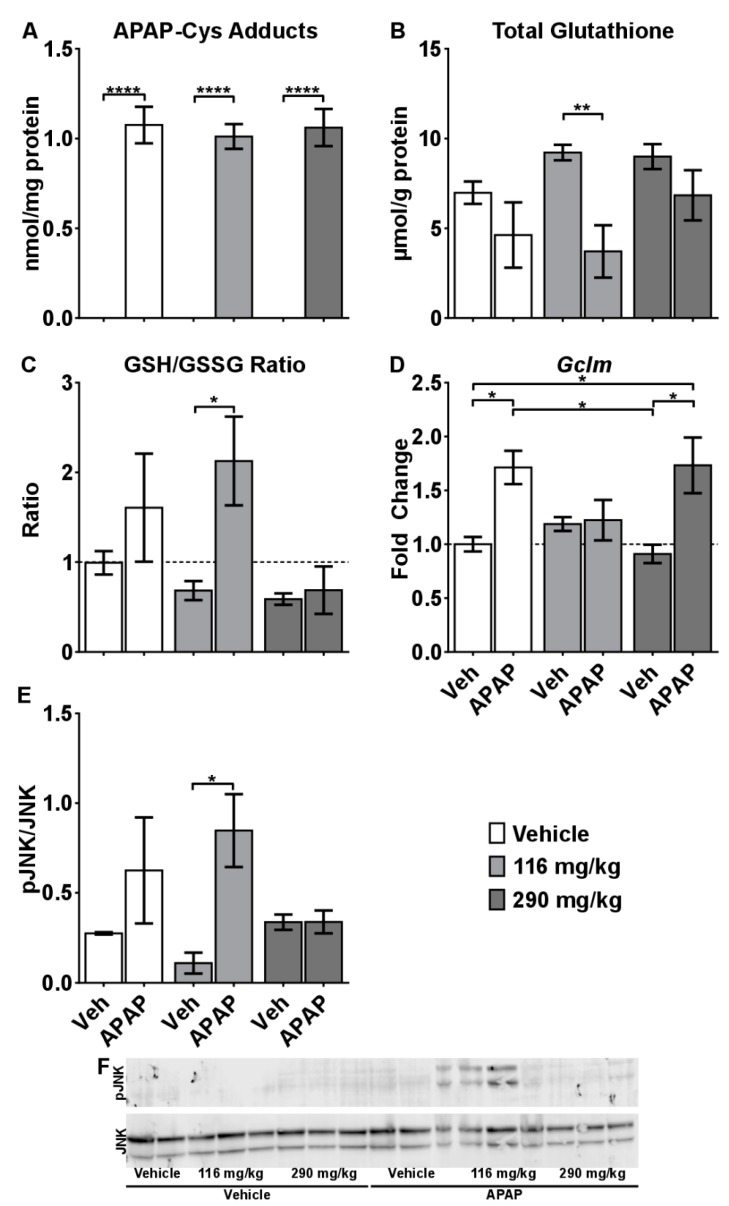
Mechanisms of CRCE/APAP-induced liver injury. (**A**) APAP-Cys protein adducts; levels of (**B**) GSH, and (**C**) GSSG/GSH; (**D**) mRNA levels of *Gclm* as measured by quantitative real-time (qRT) PCR; E) protein levels of Jnk and pJNK as measured by Western blot; and F) representative blot. Veh (*n* = 7); Veh + APAP (*n* = 7); 116 mg/kg CBD (*n* = 7); 116 mg/kg CBD + APAP (*n* = 7); 290 mg/kg CBD (*n* = 8); 290 mg/kg CBD + APAP (*n* = 8). Data presented as mean ± SEM fold changed from vehicle (* *p* < 0.05; ** *p* < 0.01; and **** *p* < 0.0001). GSSG levels were significantly affected by APAP (*p* = 0.0151), but there are no significant comparisons.

Metabolism of APAP to the reactive metabolite (*N*-acetyl-p-benzoquinone imine; NAPQI) through the CYP P450s, especially CYP2E1, is well recognized for its role in the initiation of toxicity [18]. Because of the robust increase in *Cyp2e1* mRNA levels observed in the 290 mg/kg CBD + APAP group, we hypothesized that CBD increases metabolism and bioactivation of APAP. Therefore, we measured acetaminophen protein adducts levels (APAP-CYS), a generally recognized biomarker of APAP exposure linked to the formation of toxic NAPQI. APAP-CYS were increased in the APAP treated mice. However, no difference in APAP–CYS were observed among the Veh + APAP, compared to the 116 mg/kg CBD+APAP and 290 mg/kg CBD+APAP mice (Figure 4A). Furthermore, there was no correlation between the degree of liver injury observed in the mice and the amount of APAP-CYS.

APAP depletes hepatic glutathione very early in the time course of APAP toxicity (e.g., 1 h) in the mouse, and re-synthesis occurs after 4 h [19]. Glutathione depletion causes oxidative stress and also contributes to SOS [20]. At 5 h, hepatic GSH was not statistically different in the Veh + APAP mice (although trend towards reduction was observed), but was significantly reduced in the mice receiving 116 mg/kg CBD + APAP (Figure 4B). In mice administered 290 mg/kg CBD + APAP, an unexpected and rapid re-synthesis of glutathione was observed, in which total glutathione approximated that observed in control mice (Figure 4B). Interestingly, the GSSG/GSH ratio, an indicator of liver redox status, was spiked in 116 mg/kg CBD+APAP, but not in the other groups (Figure 4C). Furthermore, the highest GSSG/GSH values–indicative of an overwhelming state of oxidative liver stress–were observed in mice characterized by the highest degree of histopathological, physiological, and biochemical end-points of liver injury (Table 2).

To further confirm these findings, we performed mRNA analysis of *Gclm*, one of the rate-limiting enzymes for glutathione re-synthesis, and determined protein levels of phosphorylated JNK, which is known to be activated by oxidative stress. Consistent with histopathological, physiological and clinical biochemistry data, significant increases in *Gclm* expression were observed in the vehicle + APAP and 290 mg/kg CBD + APAP groups, suggesting rapid re-synthesis of glutathione (Figure 4D). However, expression of *Gclm* was not induced in the 116 mg/kg CBD + APAP mice, indicating that the normal response to enhance re-synthesis of glutathione was impaired. Lastly, we observed increased JNK phosphorylation in 116 mg/kg CBD+APAP mice (Figure 3E,F). Taken together, these data are consistent with the hypothesis that 116 mg/kg CBD enhanced overall toxicity by increasing hepatic JNK activation and impairing glutathione synthesis.

## 3. Conclusions

Here, for the first time, the in vivo potential for a CBD/drug interaction resulting in liver injury has been demonstrated. Treatment of mice with clinically relevant CBD doses in CRCE for three consecutive days, followed by a challenge with APAP, resulted in the development of an SOS-like pattern of liver injury and mortality, neither of which were observed in the Veh + APAP group. Mechanistically, the observed effect was associated with an overwhelming oxidative catastrophe triggered by massive oxidative stress, rapid depletion of liver glutathione, impaired glutathione re-synthesis, and greater JNK activation. Of particular concern, this effect was observed after administration of the lower, 116 mg/kg, CBD dose in CRCE (i.e., MED = 10 mg/kg). Further studies are clearly needed to investigate this paradoxical effect and its mechanisms.

Congruent with the results of our study, emerging evidence suggests that CBD creates a significant drug interaction that could lead to serious adverse health effects, including liver injury. For instance, in a recent clinical trial, combining CBD with valproic acid led to a more robust elevation in liver enzymes, such that liver transaminases were ≥3 times the upper limit of normal in 17% of patients [21]. Our data corroborate recent clinical observations regarding CBD hepatotoxicity and caution against its casual, non-medically supervised usage with potentially hepatotoxic medications.

## 4. Materials and Methods

### 4.1. CBD Extract Characterization, Dosing Solution and Dose Calculations

Cannabidiol-rich cannabis extract (CRCE) was prepared at the University of Mississippi’s National Center for Natural Product Research by extraction of CBD rich cannabis plant material (5.61% of CBD and 0.2% THC) using hexane as the extraction solvent. The extract was then evaporated to dryness, followed by raising the temperature to 80 °C to affect complete decarboxylation of the extract.

Phytochemical analysis of CRCE showed the following: cannabidiol content–57.9%; other cannabinoids: cannabichromene–2.03%, Δ^9^-tetrahydrocannabinol–1.69%, cannabigerol–1.07%, Δ^8^-tetrahydrocannabinol–<0.01%; tetrahydrocannabivarin–<0.01%. The residual solvent was <0.5%; loss on drying–0.32%; heavy metals: lead, mercury, cadmium, and arsenic–not detected; aflatoxins: AFB_1_, AFB_2_, AGF_1_, AFG_2_–not detected.

CRCE dose was calculated based on CBD content in the characterized extract to deliver the required dose of CBD. The extract was diluted in sesame oil to prepare the gavage solution. Allometric scaling for CBD mouse equivalent doses (MED) was determined per the recommendation of Wojcikowski and Gobe which, in turn, is based upon the FDA Industry Guidance for Estimating the Maximum Safe Starting Dose in Initial Clinical Trials for Therapeutics in Adult Volunteers [22]. The scaling factor of 11.6, commonly used for mice weighing between 36–50 g, was used to calculate the MED for CBD, using the lowest recommended human maintenance dose of CBD (10 mg/kg EPIDIOLEX^®^). Thus, 1X and 2.5X MED doses consisted of 5.104 mg total CBD (116 mg/kg) and 18.45 mg total CBD (290 mg/kg respectively, delivered in 300 µL of gavage CRCE solution in sesame oil. Similar doses were used in our previous 2-week repeated dosing study on CBD and were not associated with hepatotoxicity [7]. Control mice received 300 µL of sesame oil.

### 4.2. Animals

Nulliparous female CD-1 mice were purchased from Charles River (Wilmington, MA, USA) and aged at the University of Arkansas for Medical Sciences (UAMS) Division of Laboratory Animal Medicine (DLAM) facility. We used outbred mice in order to mimic the genetic heterogeneity of humans. At 9 months of age, mice were randomly assigned to the following groups: control (*n* = 7), 116 mg/kg CBD (*n* = 7), 290 mg/kg CBD (*n* = 8), APAP (400 mg/kg; *n* = 7), 116 mg/kg CBD + APAP (*n* = 8), and 290 mg/kg CBD + APAP (n=8). APAP was purchased from Sigma (St. Louis, MO, USA; >98% purity). Mice were gavaged with CRCE for three consecutive days at 0800, followed by i.p. injection of APAP (400 mg/kg) dissolved in warm PBS on day 4. With this experimental setup, we sought to mimic conventional usage of CBD followed by ingestion of a high dose of APAP, a likely scenario given the popularity of both compounds. Animals were euthanized 5 h after APAP administration. To avoid any potential fasting-exacerbated toxicity, food and water were provided *ad libitum*. All procedures were approved by the UAMS Institutional Animal Care and Use Committee (protocol number: AUP #3701).

### 4.3. Blood Sampling and Clinical Biochemistry

Blood was collected from the retro-orbital plexus with a heparinized micro-hematocrit capillary tube (Fisher Scientific, Pittsburg, PA, USA) and placed into a 1.1 mL Z-gel microtube (Sarstedt, Newton, NC, USA). Tubes were kept on ice prior to centrifugation at 10,000 rpm for 20 min; serum samples were delivered to the Veterinary Diagnostic Laboratory at the Arkansas Livestock and Poultry Commission (Little Rock, AR, USA) for further analysis.

### 4.4. Histopathological Assessment

Liver sections were fixed in 4% formalin and processed at the UAMS Pathology Core Facility. Two independent, blinded pathologists evaluated the hematoxylin eosin sections at 20×, 40×, 100×, 200×, and 400× magnification, as described in Supplementary Materials and Methods.

### 4.5. Analysis of mRNA Levels of Major Cytochromes and Transporter Genes 

Total RNA was extracted from flash frozen liver tissue using the RNeasy Mini Kit (Qiagen, Germantown, MD, USA). Following purification, 1000 ng were reverse transcribed with the High Capacity cDNA Reverse Transcription Kit (ThermoFisher, Waltham, MA, USA). Primers were added at a final concentration of 5 µM (Supplementary Table S1). Gene expression values were calculated as fold change from control according to the _ΔΔ_CT method.

### 4.6. Analysis of the APAP Protein Adducts in the Liver Tissue

APAP-protein adducts were measured as previously described, with modifications [23,24]. Details are provided in Supplementary Materials and Methods.

### 4.7. Glutathione Analysis

Hepatic glutathione was measured using a modified Tietze assay [25] as described in Supplementary Materials and Methods.

### 4.8. Western Blot

Protein levels for JNK and pJNK were measured by western blot, as previously described [26].

### 4.9. Statistical Analysis

Data were analyzed with either Graphpad Prism 6 software (Graphpad Software, San Diego, CA, USA) or R (The R Foundation). The primary contrasts of interest were Vehicle + APAP at CBD doses of 0, 116, and 290 mg/kg for each of the measured study parameters. A 2 × 3 factorial arrangement of treatments analysis of variance model was fit to each response variable. Next, the above three contrasts were computed using a Bonferroni correction to assure a 95% family-wise confidence level. Interaction effects were evaluated using a two-way ANOVA. Bonferrroni adjusted *p*-values were also computed. Histology scores were analyzed with a Mann-Whitney test comparing vehicle versus APAP at each CBD dose. *p*-values were adjusted with a Bonferroni correction (3 comparisons for α ≤ 0.0167). Response variables were also analyzed for correlation using Spearman’s Rank Correlation due to non-continuous data in the histological analyses.

## Figures and Tables

**Table 1:** Histopathological evaluation of mouse livers using a modified Rubbia-Brandt sinusoid obstructive syndrome (SOS)-I scoring system.

	Vehicle (*n* = 7)	CBD, 116 mg/kg (*n* = 7)	CBD, 290 mg/kg (*n* = 8)	APAP (400 mg/kg) (*n* = 7)	CBD, 116 mg/kg + APAP (*n* = 8)	CBD, 290mg/kg + APAP (*n* = 8)
Sinusoidal dilation	0.0 ± 0.0	0.0 ± 0.0	0.1 ± 0.1	0.9 ± 0.4	2.0 ± 0.5 *	0.8 ± 0.5
Venous obstruction	0.0 ± 0.0	0.0 ± 0.0	0.0 ± 0.0	0.0 ± 0.0	0.0 ± 0.0	0.0 ± 0.0
Atrophy	0.0 ± 0.0	0.0 ± 0.0	0.0 ± 0.0	0.3 ± 0.2	0.6 ± 0.2	0.3 ± 0.2
Apoptosis	0.0 ± 0.0	0.0 ± 0.0	0.0 ± 0.0	0.0 ± 0.0	0.1 ± 0.1	0.1 ± 0.1
Necrosis	0.0 ± 0.0	0.0 ± 0.0	0.0 ± 0.0	0.7 ± 0.6	2.0 ± 0.6 *	0.8 ± 0.4
Microvesicular steatosis	0.0 ± 0.0	0.0 ± 0.0	0.0 ± 0.0	0.0 ± 0.0	0.0 ± 0.0	0.0 ± 0.0
Small droplet steatosis	1.0 ± 0.3	0.7 ± 0.4	0.6 ± 0.3	0.3 ± 0.3	1.0 ± 0.3	1.1 ± 0.3
Large droplet steatosis	0.0 ± 0.0	0.0 ± 0.0	0.1 ± 0.1	0.1 ± 0.1	0.0 ± 0.0	0.0 ± 0.0
Clear cell changes	0.0 ± 0.0	0.3 ± 0.3	0.8 ± 0.3	1.4 ± 0.4	0.6 ± 0.4	1.3 ± 0.5
Portal inflammation	0.2 ± 0.1	0.0 ± 0.0	0.1 ± 0.1	0.0 ±0.0	0.5 ± 0.4	0.0 ± 0.0
Lobular inflammation	0.9 ± 0.2	1.2 ± 0.3	0.8 ± 0.2	0.6 ± 0.2	1.4 ± 0.6	0.8 ± 0.2
Interface activity	0.0 ± 0.0	0.0 ± 0.0	0.0 ± 0.0	0.0 ± 0.0	0.4 ± 0.4	0.0 ± 0.0

Scores between vehicle and APAP groups at each dose of CBD were analyzed with a Mann-Whitney test and a Bonferroni correction for multiple comparisons. Significance was therefore determined at α ≤ 0.0167. Data presented as average scores for each parameter ± SEM with an * indicating a significant difference between vehicle and APAP groups at the corresponding dose of CBD. Liver samples of all experimental animals (*n* = 45) were evaluated. Details on scoring parameters are provided in the Supplementary Materials and Methods Section.

**Table 2:** List-wise format of each of the 15 unique correlations in the correlation matrix.

Row	Column	Correlation	*p*-Value
Liver-to-body weight ratio	Bilirubin	0.37	0.0210
Liver-to-body weight ratio	Total GSH	−0.17	0.3897
Liver-to-body weight ratio	GSSG/GSH	0.28	0.1465
Liver-to-body weight ratio	Sinusoidal dilation	0.42	0.0065
Liver-to-body weight ratio	Necrosis	0.56	0.0002
Bilirubin	Total GSH	−0.49	0.0043
Bilirubin	GSSG/GSH	0.48	0.0054
Bilirubin	Sinusoidal dilation	0.61	<0.0001
Bilirubin	Necrosis	0.67	<0.0001
Total GSH	GSSG/GSH	−0.69	<0.0001
Total GSH	Sinusoidal dilation	−0.75	<0.0001
Total GSH	Necrosis	−0.70	<0.0001
GSSG/GSH	Sinusoidal dilation	0.74	<0.0001
GSSG/GSH	Necrosis	0.71	<0.0001
Sinusoidal dilation	Necrosis	0.86	<0.0001

## Supplementary Materials

The following are available online, Table S1: Forward and reverse primer sequences for cytochrome P450s and UDP-gluconosyltrasferases, Figure S1: Changes in body weight and organ-to-body weight ratios after CBD ± APAP, Figure S2: Clinical chemistry parameters in the serum of experimental mice.
