# Supplementary material for: Paradoxical Patterns of Sinusoidal Obstruction Syndrome-Like Liver Injury in Aged Female CD-1 Mice Triggered by Cannabidiol-Rich Cannabis Extract and Acetaminophen Co-Administration
Source: Molecules. 2019 Jun 17;24(12):2256. doi: 10.3390/molecules24122256 (PMC6630875; doi:10.3390/molecules24122256)
Supplement: Supplementary file 1 [file molecules-24-02256-s001.pdf]

Supplemental Tables and Figures

Table S1. Forward and reverse primer sequences for cytochrome P450s and UDP-gluconosyltransferases.

	Forward	Reverse
Cyp1a1	TGCCCTTCATTGGTCACATG	CACGTCCCCATACTGCTGACT
Cyp1a2	GACATGGCCTAACGTGCAG	GGTCAGAAAGCCGTGGTTG
Cyp2b10	AAGGAGAAGTCCAACCAGCA	CTCTGCAACATGGGGGTACT
Cyp2d22	CAGTGGTTGTACTAAATGGGCT	GCTAGGACTATACCTTGAGAGCG
Cyp2e1	TCCCTAAGTATCCTCCGTGA	GTAATCGAAGCGTTTGTTGA
Cyp3a4	AAAGCCGCCTCGATTCTAAGC	ACTACATCCCGTGGTACAACC
Cyp3a11	ACAAACAAGCAGGGATGGAC	GGTAGAGGAGCACCAAGCTG
Ugt1a1	CACCTGAAGCCTCAATACCAT	CAGTCCGTCCAAGTTCCACC
Ugt1a6	ATACCATGGGAGCCAGAGTG	ACCAGAACTGTGAGGGTTGG
Ugt1a9	CTGGTTCAGCCAGAGGTTTC	TTGGCGACAATTAATCCACA
Ugt2a3	CCCAGAAGGTTTTGTGGAGA	CCACCATGTGTGATGAAAGC

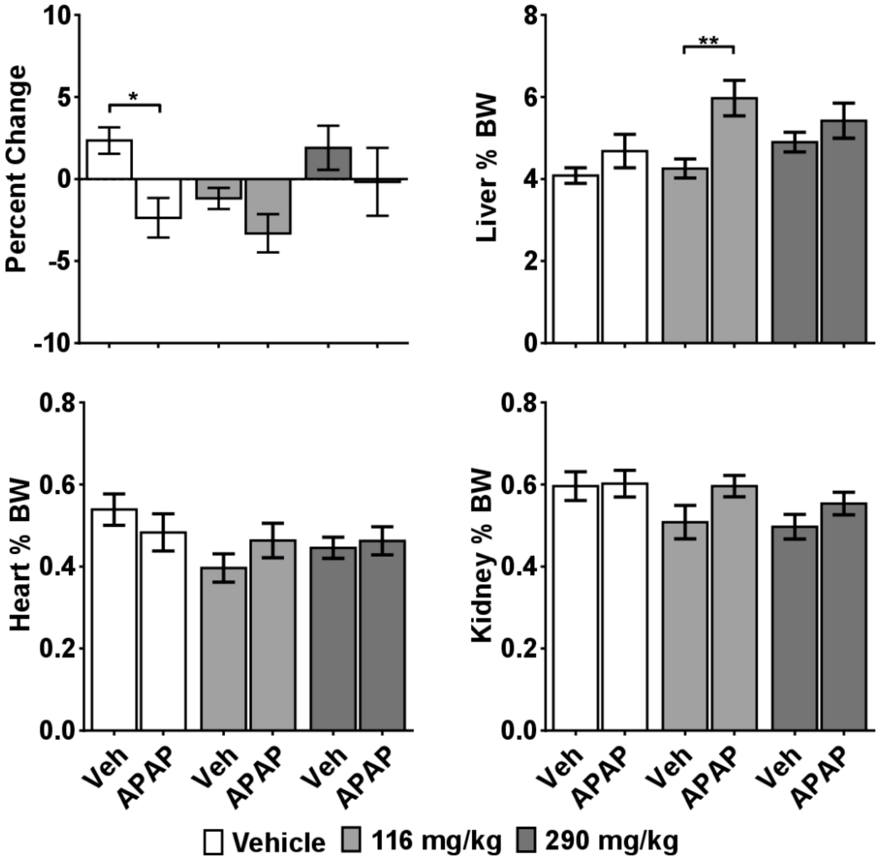

Supplemental Figure 1. Changes in body weight and organ to body weight ratios after CBD +/- APAP. A). Liver-to-body weight ratios were increased with combined treatment of 116 mg/kg CBD and APAP B), but there were no differences in either heart- C) or kidney- D) to-body weight ratios. Data presented as mean ± SEM fold changed from vehicle (*, p < .05 and **, p < .01).

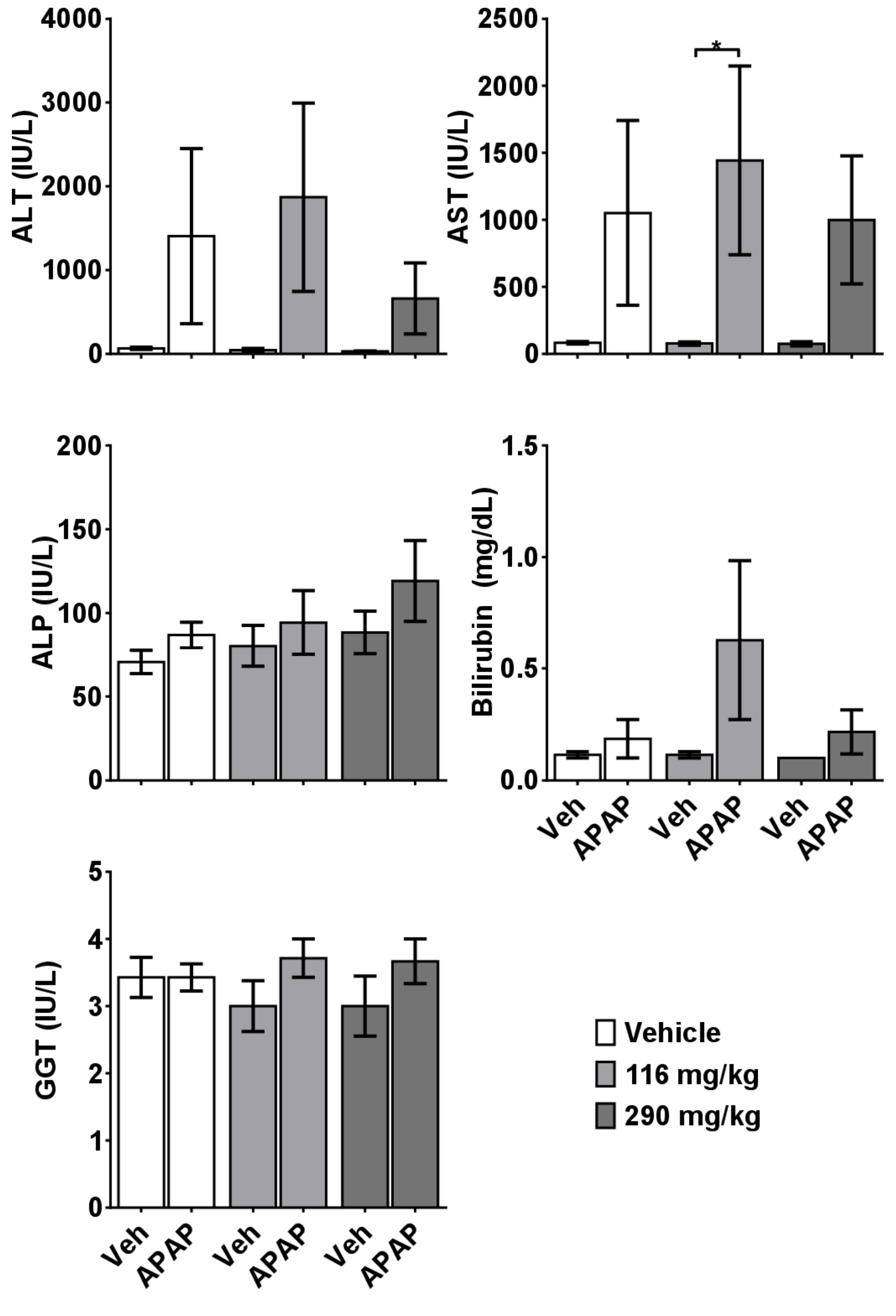

Supplemental Figure 2. Clinical chemistry parameters in the serum of experimental mice. Data presented as mean ± SEM (*, $p < .05$).

1. Supplemental Materials and Methods.

1.1 Scoring system for sinusoidal obstructive syndrome (SOS).

Scoring was performed by adapting two human scoring systems, as described in Rubbia-Brandt *et* al [1] and Stevenson *et* al [2]. Steatosis was also assessed based on a proposed NAFLD scoring system in rodent models by Liang et al [3] and additional parameters were evaluated according to the schema described below.

1. Sinusoidal dilation: 0=absent; 1=mild (centrilobular involvement limited to 1/3 of lobule); 2=moderate (centrilobular involvement extending in 2/3 of lobule); 3=severe (complete lobular involvement or centrilobular involvement extending to adjacent lobules with bridging congestion).
2. Venous obstruction: 0=absent; 1=present, partial obstruction of central venule(s); 2=present, total occlusion of central venule(s).
3. Atrophy: 0=absent; 1=present.
4. Apoptosis (single cell acidophil bodies): 0=absent; 1=present.
5. Centrivenular regional necrosis: 0=absent; 1=>0-5%; 2=>5-33%; 3=>33-66%; 4=>66%.
6. Microvesicular steatosis: 0=absent; 1=minimal (<5%), 2=mild (>5-33%), 3=moderate (>33-66%); 4=severe (>66%).
7. Small droplet steatosis: 0=absent; 1=minimal (<5%), 2=mild (>5-33%), 3=moderate (>33-66%); 4=severe (>66%).
8. Large droplet steatosis: 0=absent; 1=minimal (<5%), 2=mild (>5-33%), 3=moderate (>33-66%); 4=severe (>66%).
9. Clear cell changes: clear cytoplasm, mild cellular swelling, with or without flocculant cytoplasm or fine cytoplasmic granular contents; 0=absent; 1=minimal (<5%), 2=mild (>5-33%), 3=moderate (>33-66%); 4=severe (>66%).
10. Portal inflammation: 0=absent; 1 mild, 2=moderate; 3=severe.
11. Lobular inflammation: 0=absent; 1=minimal (1-2 foci, 20x), 2=mild (3-6 foci, 20x), 3=moderate (7-12 foci, 20x); 4=severe (>12 foci, 20x); 5=diffuse (abundant, marked panlobular).
12. Interface activity: 0=absent; 1=mild (focal, few portal areas); 2=mild/moderate (focal, most portal areas); 3=moderate (continuous, <50% of portal tracts); 3=severe (continuous, >50% of portal tracts).

1.2 Analysis of the APAP protein adducts

APAP-protein adducts were measured as previously described, with modifications [4,5]. Briefly, liver tissues were homogenized in 1x phosphate-buffered saline using a Fisherbrand Bead Ruptor homogenizer. The homogenates were then centrifuged at 12,000 g for 5 min to pellet cell debris, and two aliquots of supernatant were collected. One aliquot was passed through a size-exclusion column to purify protein. The protein was then digested by a mixture of proteases to liberate APAP-bound cysteine residues (APAP-CYS). The proteases were then precipitated with trichloroacetic acid and filtered out. Finally, APAP-CYS was measured in the filtrate using HPLC separation with electrochemical detection. The second aliquot was used for protein measurement by a bicinchoninic acid assay method, and APAP-CYS was normalized to protein.

1.3 Glutathione analysis

Glutathione was measured using a modified Tietze assay [6]. Briefly, liver tissue was homogenized in 3% sulfosalicylic acid. One aliquot was diluted in a solution containing N-ethylmaleimide (NEM) to mask reduced glutathione (GSH) and facilitate measurement of oxidized glutathione (GSSG), while another was diluted in 0.1 M HCl for measurement of total (GSH+GSSG) glutathione. After removal of NEM by solid phase extraction with a C18 column, glutathione was measured in both aliquots using a colorimetric glutathione reductase cycling detection method.

References

1. Rubbia-Brandt, L.; Lauwers, G.Y.; Wang, H.; Majno, P.E.; Tanabe, K.; Zhu, A.X.; Brezault, C.; Soubrane, O.; Abdalla, E.K.; Vauthey, J.N., et al. Sinusoidal obstruction syndrome and nodular regenerative hyperplasia are frequent oxaliplatin-associated liver lesions and partially prevented by bevacizumab in patients with hepatic colorectal metastasis. *Histopathology* **2010**, *56*, 430-439, doi:10.1111/j.1365-2559.2010.03511.x.
2. Stevenson, H.L.; Prats, M.M.; Sasatomi, E. Chemotherapy-induced Sinusoidal Injury (CSI) score: a novel histologic assessment of chemotherapy-related hepatic sinusoidal injury in patients with colorectal liver metastasis. *BMC Cancer* **2017**, *17*, 35, doi:10.1186/s12885-016-2998-2.
3. Liang, W.; Menke, A.L.; Driessen, A.; Koek, G.H.; Lindeman, J.H.; Stoop, R.; Havekes, L.M.; Kleemann, R.; van den Hoek, A.M. Establishment of a general NAFLD scoring system for rodent models and comparison to human liver pathology. *PLoS One* **2014**, *9*, e115922, doi:10.1371/journal.pone.0115922.
4. Muldrew, K.L.; James, L.P.; Coop, L.; McCullough, S.S.; Hendrickson, H.P.; Hinson, J.A.; Mayeux, P.R. Determination of acetaminophen-protein adducts in mouse liver and serum and human serum after hepatotoxic doses of acetaminophen using high-performance liquid chromatography with electrochemical detection. *Drug Metab Dispos* **2002**, *30*, 446-451.
5. McGill, M.R.; Lebofsky, M.; Norris, H.R.; Slawson, M.H.; Bajt, M.L.; Xie, Y.; Williams, C.D.; Wilkins, D.G.; Rollins, D.E.; Jaeschke, H. Plasma and liver acetaminophen-protein adduct levels in mice after acetaminophen treatment: dose-response, mechanisms, and clinical implications. *Toxicol Appl Pharmacol* **2013**, *269*, 240-249, doi:10.1016/j.taap.2013.03.026.
6. McGill, M.R.; Jaeschke, H. A direct comparison of methods used to measure oxidized glutathione in biological samples: 2-vinylpyridine and N-ethylmaleimide. *Toxicol Mech Methods* **2015**, *25*, 589-595, doi:10.3109/15376516.2015.1094844.
